# Biotic interactions govern genetic adaptation to toxicants

**DOI:** 10.1098/rspb.2015.0071

**Published:** 2015-05-07

**Authors:** Jeremias Martin Becker, Matthias Liess

**Affiliations:** 1 Department System Ecotoxicology, UFZ—Helmholtz Centre for Environmental Research, Permoserstrasse 15, 04318 Leipzig, Germany; 2 Department of Ecosystem Analysis, RWTH—Aachen University, Institute for Environmental Research, Worringerweg 1, 52074 Aachen, Germany

**Keywords:** pesticide resistance, population ecology, competition, predation, *Culex quinquefasciatus*, ecotoxicology

## Abstract

The genetic recovery of resistant populations released from pesticide exposure is accelerated by the presence of environmental stressors. By contrast, the relevance of environmental stressors for the spread of resistance during pesticide exposure has not been studied. Moreover, the consequences of interactions between different stressors have not been considered. Here we show that stress through intraspecific competition accelerates microevolution, because it enhances fitness differences between adapted and non-adapted individuals. By contrast, stress through interspecific competition or predation reduces intraspecific competition and thereby delays microevolution. This was demonstrated in mosquito populations (*Culex quinquefasciatus*) that were exposed to the pesticide chlorpyrifos. Non-selective predation through harvesting and interspecific competition with *Daphnia magna* delayed the selection for individuals carrying the *ace-1^R^* resistance allele. Under non-toxic conditions, susceptible individuals without *ace-1^R^* prevailed. Likewise, predation delayed the reverse adaptation of the populations to a non-toxic environment, while the effect of interspecific competition was not significant. Applying a simulation model, we further identified how microevolution is generally determined by the type and degree of competition and predation. We infer that interactions with other species—especially strong in ecosystems with high biodiversity—can delay the development of pesticide resistance.

## 1. Introduction

Genetic adaptation to anthropogenic toxicants has become increasingly important to the survival of populations because of environmental pollution. The failure of populations to adapt to toxicants is contributing to the biodiversity crisis [1,2]. At the same time, agricultural pests and pathogen vectors have rapidly evolved high levels of resistance to pesticides [3–6]. For example, resistance against 300 insecticides from all chemical classes commercially available has been reported in more than 500 target arthropod species worldwide, presenting challenges to conventional control strategies [4,7]. Therefore, understanding the basic processes that govern such extensive genetic adaptation is of high relevance.

The emergence of resistance to toxicants typically arises through simple mutations at a single locus. For example, the *ace-1^R^* allele, which has evolved in several mosquito species, is characterized by a point mutation that leads to a modified acetylcholinesterase (AChE), which provides high resistance against organophosphorus and carbamate insecticides [8]. Individuals carrying such a resistance allele typically display reduced fitness under non-toxic conditions, manifested, for example, in lower survival and delayed development [9,10]. This phenomenon facilitates the genetic recovery of a largely resistant population back to one dominated by susceptible individuals when toxicants are not present [11].

The fitness costs of pesticide resistance under non-toxic conditions generally increase in the presence of additional ecological stressors [12–14]. Therefore, additional stressors may hinder the development of resistance through increased fitness costs. This concept has led to a new area of research in evolutionary ecology [12]. For example, food shortages, poor food quality, toxicants not related to the developed resistance and selection by parasites or predators have increased the fitness costs of pesticide resistance in experiments under non-toxic conditions [11–18].

However, these studies did not consider the evolutionary effects of multiple interacting ecological stressors. Moreover, the effects of ecological stressors on the actual spread of resistance alleles under toxicant exposure have rarely been studied.

Here we addressed how interacting biotic stressors can affect microevolution in terms of both genetic adaptation and genetic recovery. While the primacy of biotic over abiotic stressors in driving selection has been proposed before [19], we focus on the way biotic stressors modify the adaptation to abiotic stressors such as pesticides. We hypothesized that (i) intraspecific competition promotes genetic recovery under non-toxic conditions because it enhances the fitness costs of pesticide resistance [20]; (ii) predation and interspecific competition mitigate this enhancement through a reduction of population density and hereby delay genetic recovery; and (iii) these mechanisms operate similarly on the spread of resistance under pesticide exposure.

## 2. Material and methods

We tested our predictions using selection experiments on the southern house mosquito, *Culex quinquefasciatus* Say, 1823, which is a common target species in the control of disease vectors. Mixed populations of susceptible wild-type individuals (ss) and heterozygous (sr) or homozygous (rr) individuals carrying the *ace-1^R^* resistance allele [21] were reared over six generations in a laboratory test system (see the electronic supplementary material for details). The larval density and biomass were monitored using a non-invasive image analysis system [22]. Each population was initiated with 400 larvae and an *ace-1^R^* allele frequency of 0.5 in Hardy–Weinberg equilibrium. From each population, 45 deceased adult mosquitoes per generation were genotyped using an AChE activity test [23], and the *ace-1^R^* genotype and allele frequencies were estimated. In addition, we measured the size of the genotyped mosquitoes from the first, second and sixth generations as the length of one randomly chosen wing.

Four populations were reared without species interactions; therefore, they approached carrying capacity and experienced a high level of intraspecific competition after one generation. In another four populations, approximately 10–20% of the larvae were randomly harvested twice per week using a sweep net to simulate the general effects of non-selective predation. In another four populations, we introduced 200 individuals of the water flea *Daphnia magna* at the beginning of the experiment, imposing interspecific competition on the mosquito larvae. These treatments were applied once without pesticide exposure and were repeated with another set of populations in which the mosquito larvae were exposed to 0.375 µg l^−1^ of chlorpyrifos for 24 h each generation. This concentration was chosen to dispatch greater than 50% of the homozygous susceptible larvae without causing acute effects on the heterozygous and resistant individuals (electronic supplementary material, figure S1), based on standard toxicity tests prior to the experiment [24]. *Daphnia magna* is more sensitive to chlorpyrifos than *C. quinquefasciatus* [25] and therefore was not contaminated in this experiment to ensure stable populations that act as interspecific competitors.

The data were analysed using general or generalized linear models with the software R v. 3.0.2. Mixed effects models were applied to account for repeated measurements where appropriate. The homoscedasticity and normality of the residuals were evaluated prior to the analyses, and most models were simplified to the minimum adequate model using backward selection based on likelihood ratio tests [26] (see the electronic supplementary material for details). The *p*-values were adjusted for multiple comparisons by applying either Tukey's post hoc tests or the Holm correction.

To interpret our results, we modelled the general exchange of two alleles providing unequal fitness to their carriers within an idealized, density-regulated population. We studied how the development of the allele and genotype frequencies changed when the population size was additionally affected by predation or interspecific competition under different scenarios. For this, we combined the traditional models of predation and interspecific competition from Lotka–Volterra [27–29] and applied the Hardy–Weinberg principle [30,31] to each generation to account for the mixing of genotypes during reproduction (see the electronic supplementary material for details). We considered inheritance to vary from functionally recessive to dominant, which is commonly observed for pesticide resistance and the associated fitness costs in a non-toxic environment, but excluded scenarios with overdominance that have rarely been reported [10,32,33]. The model was built in R v. 3.0.2.

## 3. Results

### (a) Reference populations

**Figure 1.**
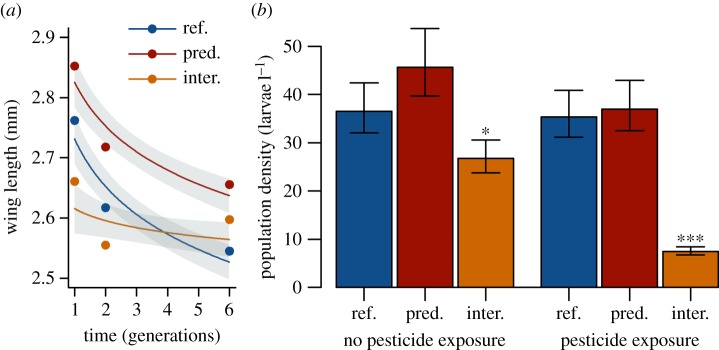
Predation and interspecific competition modify the performance of susceptible and pesticide-resistant *Culex quinquesfaciatus* mosquitoes. (*a*) The effects of interspecific competition and artificial predation on the decrease in the mean wing length of the first, second and sixth generation across all genotypes. Individuals = 3039. Populations = 4 (random factor). (*b*) Larval population density in non-contaminated and contaminated populations without further treatment (ref.), with artificial predation (pred.), and with interspecific competition by *Daphnia magna* (inter.). *N* = 4 (populations). The means ± s.e.m. are shown; the asterisks indicate significant deviations from the reference populations; *p < 0.05, ***p < 0.001.

In the reference populations, which lacked predation or interspecific competition, larval density and biomass of the mosquitoes reached an equilibrium state at approximately 41 individuals l^−1^, or 20.9 mg l^−1^ within one generation. This equilibrium indicated the carrying capacity of the populations. An overall decrease in adult body size over the course of the experiment suggested high levels of intraspecific competition among the mosquito larvae (*p* < 0.001, figure 1*a*). Exposure to chlorpyrifos did not reduce the average larval density (figure 1*b*) or biomass in the reference populations. The overproduction of young larvae, which otherwise starved during larval development, probably compensated for the mortality caused by the pesticide. Instead, pesticide exposure reduced the average abundance of adult mosquitoes in all populations from 26.6 to 21.7 individuals (*p* = 0.002). This reduction pointed to decreased developmental success because of the delayed effects of pulse contamination and the reduced performance associated with the resistance allele.

**Figure 2.**
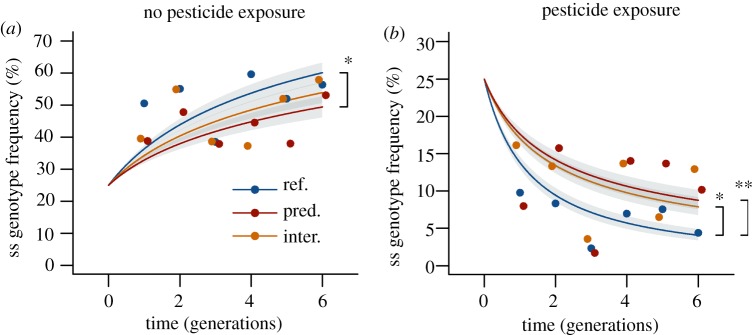
(*a*) In populations of the mosquito *Culex quinquefasciatus*, non-selective predation (pred.) and interspecific competition (inter.) delay the increase of the homozygous susceptible (ss) genotype frequency under non-toxic conditions, compared to reference populations (ref.) without species interactions. (*b*) Non-selective predation and interspecific competition also delay the decrease of the homozygous susceptible (ss) genotype frequency when the species is exposed to a pesticide. *N* = 4 for each group of populations. The points represent the mean values for each generation and were calculated using separate generalized linear models with a binomial error distribution for each generation. The lines represent the trend over all generations, which was analysed using a generalized linear mixed effects model with the log-transformed generations included as a covariate; the intercepts were fixed at the known initial genotype or allele frequency. The means ± s.e.m. are reported. The asterisks indicate significant contrasts; *p < 0.05, **p < 0.01.

The monitoring of the genotype and allele frequencies revealed a rapid process of microevolution. The populations without pesticide exposure shed the costly resistance allele, with the frequency of *ace-1^R^* decreasing from 50 to 28% over the course of the experiment (*p* < 0.001; electronic supplementary material, figure S2). In particular, homozygous susceptible individuals (ss) increased in frequency from 25 to 60% in these populations (*p* < 0.001, figure 2*a*) and displaced the other genotypes, particularly heterozygous individuals (sr). By contrast, in the populations with pesticide exposure, the frequency of *ace-1^R^* increased from 50 to 76% (*p* < 0.001; electronic supplementary material, figure S2*e*). We observed a particularly strong selection against ss. The frequency of this genotype decreased from 25 to 5% (*p* < 0.001, figure 2*b*), whereas the frequency of rr increased from 25 to 56% (*p* < 0.001; electronic supplementary material, figure S2*d*) by the end of the experiment. The frequency of sr declined more slowly than in the non-contaminated populations (*p* < 0.001; electronic supplementary material, figure S2*a*,*b*).

### (b) Predation

Predation released the mosquito larvae from high intraspecific competition, as indicated by the larger adult body size (measured as wing length; *p* = 0.005, figure 1*a*) and slightly higher fecundity, compared with the reference populations (0.78 versus 0.62 egg rafts per female, *p* = 0.053). A higher fecundity, probably combined with the reduced density-dependent larval mortality, compensated for the removal of larvae, such that the biomass and density of the larval populations remained stable (figure 1*b*). Under non-toxic conditions, ss displaced sr and rr more slowly compared to the reference populations, (*p* = 0.043, figure 2*a*). Similarly, under pesticide pressure, the selection against ss was reduced (*p* = 0.005, figure 2*b*). Additionally, the adult body size of ss individuals decreased more strongly than that of rr mosquitoes under pesticide pressure. Under non-toxic conditions, primarily the size of the rr individuals decreased (*p* < 0.001). This finding indicates that ss individuals suffered not only from acute mortality during contamination but also from reduced developmental success afterwards. By contrast, the *ace-1^R^* allele was associated with reduced developmental success under non-toxic conditions.

### (c) Interspecific competition

Interspecific competition with *D. magna* reduced the average density (*p* < 0.001, figure 1*b*) and biomass of the larval mosquito populations (20.9 versus 12.1 mg l^−1^, *p* < 0.001). This reduction was considerably greater (*p* < 0.001 for density and *p* = 0.026 for biomass) in the contaminated compared with the non-contaminated populations, probably because the strongest competitors among the mosquito larvae (ss) were debilitated as a result of pesticide exposure. This debilitation should have enabled *D. magna* to exploit more of the resources at the expense of the growth of the mosquito larvae. However, we did not observe an increase in the average density and biomass of the *D. magna* populations when they competed with the contaminated larvae. Interspecific competition generally caused the adult mosquitoes to remain small throughout the experiment; this size probably reflects their lower size limit (figure 1*a*). Interspecific competition slowed the selection against the least-adapted genotype (figure 2). This effect was present both under toxic and non-toxic conditions but was statistically significant only under pesticide exposure in which ss was displaced more slowly than in the reference populations (*p* = 0.018).

## 4. Discussion

Predation slowed both the genetic adaptation of our experimental populations to a pesticide and their genetic recovery from resistance under non-toxic conditions. Interspecific competition had the same effect but was only significant on the genetic adaptation under pesticide exposure. To ascertain the mechanisms that probably explain these results, we developed a simulation model based on the combined equations for predation and interspecific competition from Lotka–Volterra. We extended this model with the Hardy–Weinberg principle to consider microevolution (see the electronic supplementary material for details).

### (a) Intraspecific competition

In general, fitness costs can affect various fitness components that are represented by three parameters in our model: the intrinsic growth rate *r*, the carrying capacity *k* and the relative competitive strength (expressed in the competition coefficients *c*). A complete genotype and allele displacement was observed in the model when homozygous susceptible (ss), heterozygous (sr) and homozygous resistant (rr) individuals differed either in their carrying capacity or their competitive strength or both. The pace of displacement increased when a population approached carrying capacity. In this situation, intraspecific competition enhanced the contrast of fitness between the individuals of different genotypes because individuals with high fitness consume the limited resources at the expense of individuals with low fitness. This is in concordance with previous studies showing that intraspecific competition increases inequalities among individuals of various plant and animal populations [20,34]. Higher intrinsic growth rates increased intraspecific competition when populations were close to carrying capacity and expedited the process of microevolution in the model.

**Figure 3.**
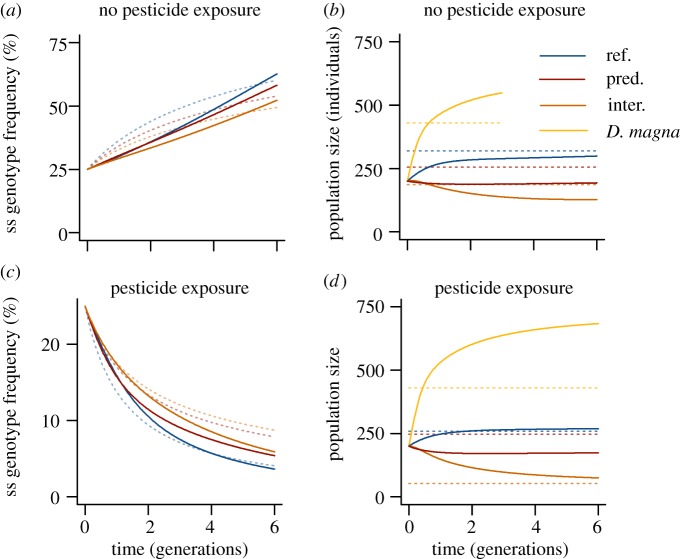
Simulation of the microevolutionary effects of predation and interspecific competition. The growth parameters of the extended Lotka–Volterra model were fitted to our experimental conditions (see electronic supplementary materials). Non-selective predation (pred.) and interspecific competition (inter.) delay the increase of susceptible (ss) individuals under non-toxic conditions (*a*) and the selection against ss individuals under toxic conditions (*c*). For comparison, the dotted lines represent the average change of the ss genotype frequencies measured in the experiment. (*b*,*d*) The simulated population sizes of the mosquitoes and of the interspecific competitor (*D. magna*). For comparison, the dotted lines represent the average population sizes over time measured in the experiment. See the electronic supplementary material, table S5 for the parameter values.

In our experiment, the reference populations were close to carrying capacity; therefore, intraspecific competition promoted the fast displacement of the costly *ace-1^R^* allele under non-toxic conditions (figure 3*a,b*). We assume that in the contaminated populations, intraspecific competition increased the displacement of ss individuals in a similar way. Toxicants considerably affect the performance and development of susceptible individuals that survived a pulse exposure [35–37], causing ss individuals to be competitively inferior to sr and rr individuals. Although acute mortality decreases the population size during contamination, survivors typically experience increased intraspecific competition afterwards because the population recovers through increased growth and reproduction [38–40].

### (b) Predation

Predation reduces the abundance of the prey population and consequently decreases intraspecific competition. In our experiment, such competitive release delayed the displacement of the least-adapted genotype, i.e. rr under non-toxic conditions and ss under toxicant pressure.

Predation significantly affected only the frequency of the ss genotype whereas we did not find significant effects on the other genotype frequencies nor on the allele frequencies. We expected this result because previous studies showed that the overall fitness costs associated with *ace-1^R^* under non-toxic conditions were mostly dominant [41–43]. Therefore, in our non-contaminated populations, selection acted primarily between ss individuals on the one hand (contributing only to the frequency of the susceptible allele) and sr and rr individuals on the other hand (contributing to both the susceptible and the resistant allele). In this situation, any environmental factor that reduces selection pressure most strongly affects the frequency of ss. Similarly, in the contaminated populations the fitness of sr and rr were more similar than that of ss. This is because the pesticide concentration used considerably affected only ss but neither sr nor rr (electronic supplementary material, figure S1). Again, a reduction of selection pressure most strongly affects the frequency of ss in this situation. The assumption of dominant fitness costs under non-toxic conditions and of dominant resistance under toxic conditions is further supported by our observation that the frequency of sr decreased faster under non-toxic conditions (electronic supplementary material, figure S2): under toxic conditions, sr decreased as a consequence of the Hardy–Weinberg principle when the allele frequencies departed from 0.5 [44]. By contrast, under non-toxic conditions, selection contributed to the decrease of sr.

The delay of microevolution through predation could be reproduced in our model (figure 3*a,b*), although the effect size under toxic conditions was smaller than observed in the experiment. However, the model did not consider that intraspecific competition increases the sensitivity of susceptible organisms to toxicants [33,45]. Hence, in real populations, selection against ss individuals may increase even more strongly with population density than predicted by our model.

**Figure 4.**
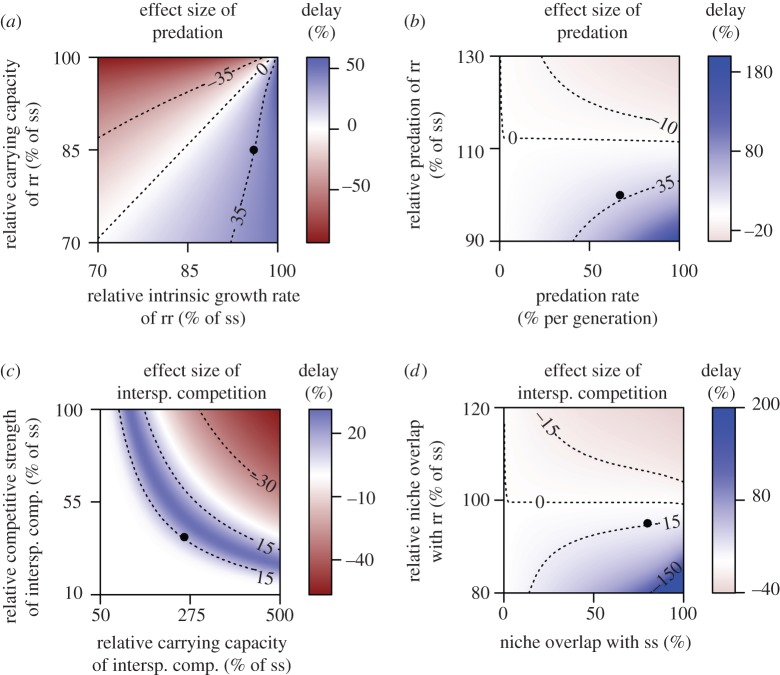
Sensitivity analysis of the simulation model. The effect sizes of predation and interspecific competition on microevolution were measured in % additional time until the genotype frequency of the susceptible wild-type reached 0.99 under non-toxic conditions. Negative effect sizes represent an acceleration of microevolution. The parameters given on the axes were varied whereas all other parameter values were held constant. The black dots indicate the parameter values that represent our experimental conditions and were used in figure 3*a,b*. (*a*) The delay through predation increases with the ratio of the fitness costs on the intrinsic growth rate and the carrying capacity and (*b*) increases with the predation rate, but decreases with the preference of a predator for individuals carrying the resistance allele. (*c*) The delay through interspecific competition is the largest at an intermediate carrying capacity and competitive strength of the competing species. (*d*) The delay through interspecific competition increases with the degree of niche overlap and the imbalance of niche overlap between wild-type individuals and pesticide-resistant individuals.

The model shows further that the evolutionary effects of non-selective predation actually depend on the life-history traits that are affected by fitness inequalities. If an allele is associated with a reduced carrying capacity or reduced competitive strength, predation delays its displacement. By contrast, predation promotes allele displacement if the allele predominantly decreases the intrinsic growth rate (figure 4*a*). If the allele affects all three parameters rather equally, predation delays microevolution. Because the fitness costs of pesticide resistance alleles usually affect various traits simultaneously [15,43,46], we therefore conclude that in most cases non-selective predation should delay microevolution.

In contrast with our experiment, natural predators may selectively feed on individuals with reduced fitness and promote microevolution [14,47]. Our model shows that such selection may actually accelerate microevolution if the preference of the predator for the prey with reduced fitness exceeds a threshold at 12% given the scenario from our experiment (figure 4*b*). By contrast, at lower preference as realised in our experiment, the delay of microevolution through the release from intraspecific competition is the most important effect. Even if our method of harvesting might have slightly favoured fitter individuals with a higher escape swimming activity, this mild selection pressure is therefore unlikely to considerably affect the observed effects of artificial predation.

### (c) Interspecific competition

Interspecific competition partly replaces intraspecific competition, particularly when populations are close to the carrying capacity. During the growth phase, it adds to the overall competition pressure faced by a population. Similar to intraspecific competitors, interspecific competitors consume resources at the expense of the genotype with the lowest fitness. Therefore, interspecific competition promotes microevolution in our model when the carrying capacity and the relative competitive strength of the interspecific competitor are high (figure 4*c*). By contrast, in the experiment we observed that interspecific competition slowed the genetic adaptation to a pesticide (and also the genetic recovery under non-toxic conditions, although this effect was not significant). This can be explained only if we assume that interspecific competitors interact more strongly with adapted individuals with high fitness.

**Figure 5.**
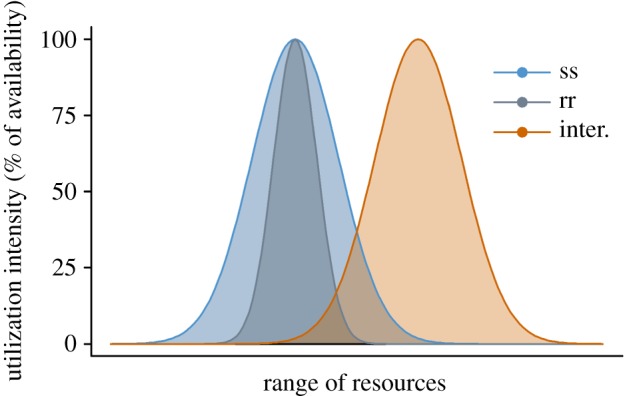
Hypothetical resource utilization of susceptible (ss) and pesticide-resistant (rr) individuals of a species. The area under each curve represents the competitive strength of the genotype. When fitness costs narrow the ecological niche of the rr individuals, interspecific competitors (inter.) with a different resource preference affect ss individuals more strongly than rr individuals.

Considering the niche theory, we suggest that this mechanism is a common case and explains our experimental results: coexisting competitors are characterized by niches that partially overlap. Genotypes with a reduced niche width are less affected by interspecific competition (figure 5). Fitness costs narrow the fundamental niche of individuals carrying the resistance allele: several studies have shown that fitness costs associated with resistance to toxicants increase under challenging conditions in which a species encounters the edge of its ecological niche [9,13,17]. For example, insects resistant to Bt toxins were found to suffer a disproportionally high mortality when grown on a suboptimal food source under non-toxic conditions [13,17]. Therefore, we expect that interspecific competition primarily affects individuals with high fitness and this way delays microevolution.

Under this assumption, interspecific competition delays microevolution in our model (figure 3). The niche of the interspecific competitor could overlap most strongly with the smaller niche of the inferior genotype only if there is a very large overlap in the resources used by the two species. In this case, our model predicts that interspecific competition accelerates microevolution (figure 4*d*). However, this situation is expected to be rare in natural communities because insufficient niche separation is prevented by competitive exclusion [20].

In our experiment, we estimated that the resources used by *C. quinquefasciatus* and the competing *D. magna* overlapped by 80% (see the electronic supplementary materials), allowing coexistence for several generations. In the non-contaminated populations, interspecific competition restricted the fitness advantage of ss and thus delayed the displacement of sr and rr. Conversely, in the contaminated populations, rr was expected to have a broader resource spectrum and to be competitively superior to ss because toxicants confine the niche breadth of susceptible organisms [48]. For example, Bourguet *et al.* [33] showed that under high pesticide stress in which resistance was functionally recessive, the mortality of sr larvae increased more than that of rr larvae with water depth, i.e. the diving time for food search. Thus, it is likely that after the moderate pesticide stress in our experiment, *D. magna* competed predominantly with the well-performing sr and rr individuals in the water column and therefore delayed the displacement of the ss individuals with reduced diving ability.

In this experiment, we contaminated only the mosquito larvae but not the interspecific competitor, reflecting the scenario of a highly selective pesticide. If competing species are similarly affected by a broad-spectrum pesticide, the niche breadth of each species is reduced to their optimum resources. This may increase the observed evolutionary effect of interspecific competition if the competitor shares its optimum resources with the resistant mosquito larvae, as was presumably the case in our experiment. Conversely, the evolutionary effect may decrease if the niches of both species overlap only slightly and therefore separate under toxic conditions.

## 5. Conclusion

**Figure 6.**
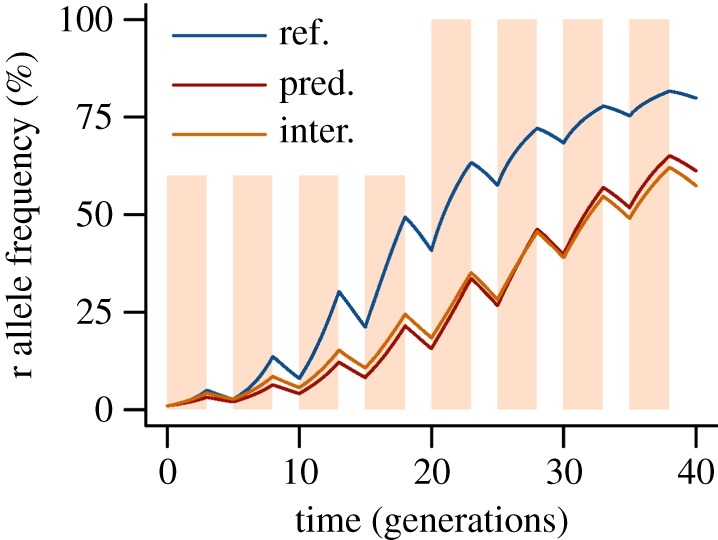
Simulation of the spread of a resistance allele r in a population alternately treated with a pesticide for three generations (red bars), while the subsequent two generations can genetically recover. The initial genotype frequencies were ss = 98%, sr = 1.99% and rr = 0.01% With the same parameter values applied as in figure 3*a*, predation (pred.) and interspecific competition (inter.) delay the increase of pesticide resistance.

We observed that non-selective predation and interspecific competition slowed microevolution within a population, delaying both the genetic adaptation to a pesticide and the genetic recovery after pesticide exposure. This delay can be best explained by a reduction of intraspecific competition, which acts as a driver of microevolution. The size and direction of the microevolutionary effects of predation and interspecific competition depend on the life-history traits that are affected by fitness costs; these effects were small in our experiment but accumulate when acting over many generations. A common scenario for pest and vector species is a low initial frequency of a resistance allele and alternating selection pressure from pesticide treatments and subsequent genetic recovery [3,49]. With the parameter values from our experiment, our simulation model demonstrates that predation and interspecific competition considerably delay the spread of the resistance allele in such a scenario (figure 6). Here the overall pace of microevolution represents a balance of increasing resistance during contaminated generations and decreasing resistance during non-contaminated generations. A small delay of both processes considerably prolongs the time until the resistance allele becomes fixed within a population. Hence, species interactions—especially strong in diverse communities—may support pesticide resistance management, an ecosystem service of biodiversity not considered so far.

## Supplementary Material

ESM Methods

## Supplementary Material

ESM Figures

## Supplementary Material

ESM Tables

## Data accessibility

The experimental data and the script of the simulation model have been deposited at Dryad Digital Repository (http://dx.doi.org/10.5061/dryad.vr7km).
