# Supplementary material for: Biotic interactions govern genetic adaptation to toxicants
Source: Proc Biol Sci. 2015 May 7;282(1806):20150071. doi: 10.1098/rspb.2015.0071 (PMC4426622; doi:10.1098/rspb.2015.0071)
Supplement: ESM Methods [file rspb20150071supp1.pdf]

Electronic Supporting Material: Detailed Methods

Test Animals

The populations of *C. quinquefasciatus* used in the experiment were derived from a colony of the SR strain, which is homozygous for the *ace-1*^{R} resistance allele, and from a colony of the susceptible SLAB strain, which shares the same genetic background [1]. The *ace-1*^{R} allele encodes a modified acetylcholinesterase (AChE) that is insensitive to organophosphates and carbamates [2]. The strains were provided by University Montpellier II and were cultured with a minimum of approximately 450 adults per generation, without pesticide contact for at least two generations. The larvae developed in Elendt M4 medium [3] with constant aeration in white plastic trays and were allowed to feed ad libitum on a 1:1 mixture of stinging nettle powder and ground dog biscuits. The adults were cultured in 60x53x53 cm cages and were provided with sponges soaked in glucose, fructose and honey. They received defibrinated cattle blood (Fiebig Animalblood Products, Idstein-Niederauroff, Germany) four times per week in a small bag composed of stretched Parafilm®. To stimulate feeding, the blood was warmed to 36°C for 30 min at the end of each night using a heating foil controlled by a thermo-timer. Additionally, human breathing air was repeatedly provided through a tube close to the blood bag. The cultures were developed at 23±1°C and 60±10% humidity under a 12:12 h LD light regime.

Toxicity Tests

Prior to the main experiment, the levels of resistance in the different genotypes were assessed following the OECD guidelines for acute immobilisation testing [3]. By derogation from this test described for *Daphnia magna*, we contaminated mosquito larvae younger than 24 h with a

series of concentrations of the organophosphate chlorpyrifos and counted the completely immobilised individuals after 24 h. Specifically, 10 mg of neat chlorpyrifos (Sigma-Aldrich, St. Louis, MO, U.S.A.) was dissolved in 5 mL of DMSO and diluted in M4 medium to the following concentrations: 6.6, 8.3, 10.4, 13.0, 16.3, 20.3 and 25.4 µg/L for the rr larvae, and 4.2, 5.2, 6.6, 8.2, 10.2, 12.8 and 16.0 µg/L for the sr larvae. The ss larvae were tested repeatedly using several of the following concentrations: 0.045, 0.076, 0.130, 0.137, 0.191, 0.221, 0.240, 0.268, 0.300, 0.375, 0.469, 0.525, 0.586, 0.638, 0.735, 1.029 and 1.084 µg/L. Dose-response models were fitted in R 3.0.2 using the drc package 2.3-7. On average, 66.6% of the ss larvae were immobilised at 0.375 µg/L, whereas the same concentration showed no effect on the other genotypes (see figure S1 in the electronic supplementary material). This concentration was used for contamination in the main experiment. For comparison, the test was also performed with neonates from 3 to 4 week-old *Daphnia magna* (LC50 = 0.428 µg/L ± 0.12 95% C.I.).

Experimental Procedures

Prior to the experiment, females of each strain were crossed with males from the other strain to obtain heterozygous offspring (F1). Offspring from both reciprocal crosses were bred separately to create the F2 generation. The experiment consisted of 24 populations that were initiated between 03.12.2011 and 30.12.2011 (day 1) because the number of available larvae at each day was restricted. Every population started with 200 randomly selected F2 larvae from each cross; therefore all the populations consisted of 400 individuals in Hardy-Weinberg equilibrium at the beginning of the experiment. The larvae of each population were equally divided into two glass cylinders with an inner diameter of 20 cm and a height of 20 cm; the cylinders were filled with 3 L of M4 medium. The larvae were introduced progressively to avoid the exceptionally high density stress caused by synchronised development at the

beginning of the experiment. On day 1, 100 first-instar larvae were introduced to each cylinder, and on days 8 and 15, another 50 first-instar larvae were added. Prior to the introduction, the larvae were placed in 200 mL of M4 medium without food for 24 h. In half of the populations, the larvae were contaminated with 0.375 µg/L of chlorpyrifos during this time. After introduction to the cylinders, 4,825 µL of a food solution (a 1:1 mixture of ground stinging nettle and dog biscuits in 1 g/L of distilled water) was added to each cylinder on days 1 and 3. Subsequently, the cylinders received 2,412.5 µL of the food solution three times per week. This type of food has been shown to support the coexistence of *Culex* and *D. magna* in previous competition studies [4, 5], and the optimal amount of food was inferred from pilot studies. The medium was aerated for 5 min three times per day with an aquarium pump, and 2.75 L of the medium was renewed every week.

In four of the contaminated and non-contaminated populations, the larvae were allowed to develop undisturbed. In another eight populations, we simulated non-selective predation by harvesting approximately 10 – 20 % of the larvae with a small sweep net twice per week. Before harvesting, the medium was stirred with a spoon to randomise predation. In the remaining eight populations, we added 25 adults of different ages (1 - 3 weeks) and 75 neonates of *D. magna* to each cylinder on day 1.

Three times per week, pupated mosquitoes from both cylinders containing the same population were counted with a glass pipette and reunited from a covered glass vessel filled with 20 mL of M4 medium. Every day, the emerged adults were released into a cage with the dimensions of 30 x 17 x 10 cm in which they had free access to a sponge soaked in a 10% glucose solution and an uncovered vessel containing M4 medium for drinking and oviposition. Additionally they received cattle blood as described above. Egg rafts were counted three times per week and transferred to 200 mL of M4 medium with 100 µL of Liquizell starter food (Dohse Aquaristik KG, Grafschaft-Gelsdorf, Germany) for the hatchlings. After two days, the young larvae of the contaminated populations were treated

with 0.375 µg/L of chlorpyrifos for 24 h. Subsequently, the larvae of each population were again divided between two glass cylinders. Thus, the mosquito larvae from each generation were contaminated once. *D. magna* was comparably susceptible to chlorpyrifos but was not contaminated, to simulate the effects of a selectively acting pesticide. After 25 weeks (approximately six generations), the larval culture was stopped, but the adult populations were further supplied until extinction. The temperature, humidity and light regime were similar to those experienced by the colonies of the mosquito strains.

Data Collection

The density and biomass of the mosquito larvae and daphnids were monitored two times per week using a system for image analysis described by Foit et al. [4]. The deceased adults were collected three times per week and their sex was determined based on their mouthparts before storage at -18°C for further analyses.

The development of the mosquitoes was desynchronised; therefore, overlap of the generations occurred. However, to estimate the shift in genotype frequencies between several generations, we considered mosquitoes from 4 consecutive weeks to belong to the same generation, among which only mosquitoes from the first 3 weeks were analysed to reduce generation overlap. In a preliminary experiment, the mosquitoes showed an average generation time of 4 weeks, with most of the males dying between weeks 5 and 7 and most of the females dying between weeks 7 and 9. Therefore, we pooled all the deceased males from weeks 5 to 7 as part of the first generation. Males from the following week, which was the time of maximum overlap between the generations, were excluded from further analyses, whereas males from weeks 9 to 11 were pooled as the second generation, and so on. Similarly, females that died between weeks 7 and 9 were pooled as the first generation, and those dying between weeks 11 to 13 were grouped as the second generation. The wing length, i.e., the distance from the tip to the

alula notch of one randomly chosen wing of individuals from the first, second and sixth generations, was measured using the AxioVision40 V.4.8.1.0 software on a Discovery.V20 microscope (Carl Zeiss Microscopy GmbH, Oberkochen, Germany).

The *ace-1* genotype of the adults was determined post mortem based on the AChE activity they presented at different diagnostic pesticide concentrations, as described by Bourguet *et al.* [2]. By derogation, we homogenised the mosquitoes in a double volume of sodium phosphate to allow a second trial to be performed in case of failure, and the concentration of DTNB used was 4x higher than that previously described. We first added the DTNB solution and the diagnostic pesticide solutions to the wells of a microplate prior to the addition of the mosquito sample and acetylthiocholine. The AChE reaction was measured over 10 min at 405 nm on a SpectraMax 250 plate reader (Molecular Devices, Sunnyvale, CA, U.S.A.). We calculated the ratio of the reaction rates at different propoxur concentrations using the equation $r = (A2/A1) * (A3/A1)$ in which A1 represents the AChE activity without propoxur, whereas A2 represents the activity at a low propoxur concentration, and A3 represents the activity at a high propoxur concentration. An $r < 0.05$ indicates a susceptible genotype, whereas $0.05 \geq r < 0.2$ indicates a heterozygous genotype, and $r \geq 0.2$ indicates a resistant *ace-1* genotype. When A1 was < 2, the basic AChE activity was too low for effective genotyping, and the sample was omitted from further analyses. In this manner, we accurately identified the phenotypes corresponding to the genotypes of 94% of a 128 reference samples from the original mosquito strains and their crosses. In each population, 45 randomly selected individuals from each generation were genotyped.

Data Analysis

All the analyses were performed using the software R 3.0.2. The data were checked for heteroscedasticity, non-normality of the residuals and overdispersion based on both graphical

methods and statistical tests, as recommended by Crawley [6]. Generalised (GLM) instead of general linear models were used if they improved the model diagnostics. All the models were simplified to the minimum adequate model using backward selection; the decision for the removal of a term was based on likelihood ratio tests of the change of deviance [6]. When a model did not include interactions, the reported p-values refer to the contrasts of main effects that were obtained from a Tukey post-hoc test using the multcomp package 1.3-1. When interactions were present, we report selected simple effect contrasts of interest to describe means or selected interaction contrasts to describe the interaction. The contrasts were analysed using the phia package 0.1-5, and the p-values were adjusted for multiple comparisons using the Holm correction. All the reported contrasts were covered by a global effect with $p < 0.05$.

The effects of contamination and additional stressors on the mean number of egg rafts per female were analysed using a two-way ANOVA. The effects of contamination and additional stressors on the size of the adult mosquito population were analysed using a linear mixed-effects model (LMM) with population as a random factor to account for repeated measurements.

To analyse the effects of contamination and additional stressors on the larval density and biomass, the moving average from three consecutive sampling dates was calculated for each test cylinder, as suggested by Foit et al. [4]. The average larval density and biomass of all the sampling dates was then analysed using a LMM (for biomass) or a generalised linear mixed-effects model (GLMM) with a Gamma residual distribution and an inverse link function (for density, figure. 1*b* and table S1). Populations were included as a random factor to account for repeated measurements. Test cylinders were nested in populations to avoid pseudoreplication because the cylinders of the same population were not considered to be independent.

The effects of contamination and additional stressors on the shifts in allele and genotype frequencies over the generations were analysed using a GLMM with a binomial residual

distribution and a logit link function (table S2). Populations were included as a random factor to account for repeated measurements. Generations were included as a continuous fixed-effect variable and log-transformed to improve the model fit. The intercepts were fixed at the known initial genotype or allele frequencies (figure 2). This was achieved by replacing all the main effects by a defined offset. Additionally, we analysed the effects of contamination and additional stressors for each generation separately, using GLMs with a binomial residual distribution and a logit link function. To achieve better comparability between the generations, the means obtained from the maximal models are presented in figure 2, but the effects marked as significant were confirmed in minimum adequate models as described above.

The effects of the genotype, contamination, additional stressors, sex and time of the experiment (expressed in generation times) on wing length were analysed using a LMM. Generations were treated as a continuous variable and log-transformed to improve the model fit (table S3). To account for repeated measurements, populations were included as a random factor in the models for genotype and allele frequencies and for wing length. Mixed-effects models were built using the lme4 package 1.0-5 for R.

Simulation Model

We modelled the potential dynamics of the genotype and allele frequencies within an ideal sexually reproducing, diploid population. The model was built in R 3.0.2 and consists of two processes that were implemented at each time step: the first part concerns the growth of the homozygous susceptible (ss), heterozygous (sr) and homozygous resistant (rr) subpopulations. The second part concerns the mixing of alleles through sexual reproduction.

For the growth part, we combined the models of Lotka-Volterra for predation and interspecific competition; e.g., the growth of ss was described with the following formula:

$$\frac{dN_{ss}}{dt} = r_{ss} * N_{ss} \frac{K_{ss} - N_{ss} - c_{ss,sr} * N_{sr} - c_{ss,rr} * N_{rr} - c_{ss,i} * N_i}{K_{ss}} - a_{ss} * N_{ss} * N_p$$

For each time step, dt, the subpopulation increases its size, N_{ss}, according to its intrinsic growth rate, r_{ss},, but is more limited by competition among its individuals the more closely it approaches its carrying capacity, K_{ss}. Growth is further limited by competition with the other subpopulations and an interspecific competitor i, depending on their competition coefficients, $c_{ss,sr}$, $c_{ss,rr}$ and $c_{ss,i}$, and their (sub)population sizes, N_{sr}, N_{rr} and N_i. The competition coefficients describe the relative competitive effect of those subpopulations on the first subpopulation: for $c_{ss,sr} < 1$, competition by sr limits the growth of ss less than competition within ss, whereas for $c_{ss,sr} > 1$, competition between the two genotypes is more important than competition within ss. Growth is additionally reduced by predation. This effect increases with the attack rate of the predator on the subpopulation, a_{ss} (% reduction of N_{ss} by a single predator), and with the (sub)population sizes of the prey and of the predator N_p. We modelled the population size of the predator according to Lotka-Volterra:

$$\frac{dN_p}{dt} = (f_{ss} * a_{ss} * N_{ss} + f_{sr} * a_{sr} * N_{sr} + f_{rr} * a_{rr} * N_{rr} + f_i * a_i * N_i) * N_p - q * N_p$$

The growth of the predator population, N_p, is determined by its attack rates, a, the fecundity per prey, f, the prey subpopulation sizes, N, and the intrinsic mortality rate of the predator, q. Based on these differential equations, the (sub)population size of e.g., ss after the growth part, N'_{ss}, was calculated as follows:

$$N'_{ss} = N_{ss} + dt * \frac{dN_{ss}}{dt}$$

Unlike different species, individuals of different genotypes can mate and produce offspring of other genotypes. Therefore, in the second part, we applied the Hardy-Weinberg principle to the new genotype frequencies at each time step. For simplicity, we assumed that preconditions of the Hardy-Weinberg principle, apart from selection, which was concerned in the growth part, were met (random mating, no genetic drift, no migration or mutation).

Within a single time step *dt* = 1/n generation times, 1/n of all individuals reproduce and die. Therefore, *dt* describes the portion of individuals in each subpopulation that switch from one generation to the next. The Hardy-Weinberg principle applies only to these reproducing individuals. Hence, the subpopulation size of e.g., ss after the mixing of alleles, N''_{ss}, is composed of two terms:

$$N''_{ss} = (1 - dt) * N'_{ss} \quad + \quad dt * (F'_s)^2 * (N'_{ss} + N'_{sr} + N'_{rr})$$

The portion of individuals who do not reproduce during the time step (1 – *dt*) normally contributes to the size of their subpopulations. In contrast, the portion of individuals who reproduce during the time step (*dt*) exchange alleles. Following the Hardy-Weinberg principle, they contribute to the different subpopulation sizes depending on the frequencies of the susceptible (F'_s) and resistance (F'_r) alleles. In contrast to N''_{ss}, the subpopulation of sr after the mixing of alleles was calculated as follows:

$$N''_{sr} = (1 - dt) * N'_{sr} \quad + \quad dt * 2 * (F'_s) * (F'_r) * (N'_{ss} + N'_{sr} + N'_{rr})$$

Parameterisation

The model was parameterised to meet our experimental conditions and was run for 6 generations, in time steps of 0.01 generation times. All the parameter values are summarised in table S5.

Initial Conditions

The initial subpopulation sizes of the different genotypes of *C. quinquefasciatus* were set to $N.start_{ss} = N.start_{rr} = 50$ and $N.start_{sr} = 100$, resembling the experimental population sizes at the beginning of the experiment. No introduction of additional individuals after 7 or 14 days was considered in the model because in contrast to the experiment, the populations are considered to be completely desynchronised in the simulation and therefore start to propagate

from the first day on. This early propagation resembles the introduction of additional individuals during the first weeks of our experiment.

To simulate the reference populations without predation or interspecific competition, we excluded the effects of interacting species by setting their initial population sizes to $N.start_p$ = $N.start_i$ = 0. To simulate the populations that were harvested, the initial population size of the predator was set to $N.start_p$ = 1, representing the experimenter. To simulate the populations with interspecific competition, the initial population size of the interspecific competitor *D. magna* was set to $N.start_i$ = 200.

Parameter Values for the Different Genotypes

We first estimated the parameter values for the average intrinsic growth rate, *r*, and the carrying capacity, *K*, of our experimental mosquito populations under non-toxic conditions. These parameter values for *C. quinquefasciatus* as a whole were considered to represent those for the ss genotype. Therefore, K_{ss} was set to the average size of the larval populations under non-toxic conditions when they had reached carrying capacity during the generations 2 – 6. r_{ss} was obtained from Mulatti et al. [7] who estimated a maximum intrinsic growth rate of 2.0 generation^{-1} in natural populations of the closely related species *C. pipiens* when the population density was low. The relative competitive strength of ss mosquitoes, $comp_{ss}$, under non-toxic conditions was set to 1 as the reference. The relative competitive strength did not directly occur as a parameter in the model but was used to calculate the competition coefficients, *c*, for the relationships between the genotypes. For instance, the competition coefficient $c_{rr,ss}$ describing the growth limiting effect of ss on rr individuals complies with the ratio: $\frac{comp_{ss}}{comp_{rr}}$.

We estimated the deviations of *K*, *r* and *comp* for the sr and rr genotypes by fitting the simulated genotype frequencies to the experimentally observed genotype frequencies. For that purpose, we varied the parameter values for the rr mosquitoes, r_{rr}, K_{rr} and $comp_{rr}$,

independently from one another from 75 – 100 % of those for ss mosquitoes, in steps of 5 %. The parameter values for the sr mosquitoes depended on the dominance level of the fitness costs of resistance for this parameter, *dominance*, and were calculated as in the following example: $r_{sr} = r_{ss} - (r_{ss} - r_{rr}) * dominance.r$. The dominance of the fitness costs was also varied independently for each parameter from 60 – 100 % in steps of 20 %. With each combination of parameter values we simulated three scenarios, without species interactions and with predation or interspecific competition. The resulting genotype and allele frequencies at each generation were compared to the average frequencies observed in the experiment. We searched for the parameter combination that produced the least sum of squared deviations from the experimental results for all frequencies in all scenarios. To increase the preciseness of our estimates, the process was repeated starting with the combination of parameter values that produced the best fit and decreasing or increasing each parameter value independently by up to 2 % in steps of 1 %.

r_{rr}, K_{rr} and $comp_r$ were considered to be the same under toxic and non-toxic conditions because of the high level of pesticide resistance. To estimate r_{ss}, K_{ss}, and $comp_{ss}$ under toxic conditions, we varied the values for these parameters from 75 – 100 % of those of rr individuals in steps of 5 %. The dominance of the fitness costs from pesticide exposure was also varied as described above, and the simulated genotype and allele frequencies were compared with the experimental results from the populations under toxic conditions. For more precise estimates, the process was repeated starting with the combination of parameter values that produced the best fit and by decreasing or increasing each parameter value independently by up to 2 % in steps of 1 %.

Parameter Values for Predation

The fecundity, f, and mortality, q, of the predator were set to $f = q = 0$, representing the stable population size of 1 experimenter. In the experimental populations, the larvae pupated on

average after 2.5 weeks and we harvested approximately 20 % of the larvae twice per week. Therefore, 80 % of the individuals escaped the artificial predation 5 times during their larval development, and we set the attack rate of the predator to $a = 1 – 0.8^5 = 0.67$. This attack rate was assumed to be similar for all genotypes because we randomised the process of harvesting.

Parameter Values for Interspecific Competition

The intrinsic growth rate of the interspecific competitor *D. magna*, r_i, was estimated from a life table obtained from daphnids that were grown individually for three weeks in 80 mL of M4 medium with an optimised supply of the food solution described above (Tab S2). Each day, the progeny was counted and removed to avoid competition. The fecundity of the survivors in week 4 was not measured but was estimated based on observations from the main culture, because late reproduction contributes very little to r [8]. With these data, we estimated r_i from the equation $\sum e^{-r\,x}\, l_x\, m_x = 1$ as described by Birch [8], in which l_x represents the probability of a cohort to survive to the age x, and m_x represents the average number of female offspring per female during that age. Because the simulation treats time in generation times, we also measured x in generation times of *C. quinquefasciatus*. Based on observations during the experiment, we set 1 generation time = 4 weeks and obtained $r_i = 10.10$ generations^{-1} of *C. quinquefasciatus*.

To estimate the carrying capacity of *D. magna* without interspecific competition, K_i, we plotted the population size of *C. quinquefasciatus* at each date against those of *D. magna*. Because the intercept of this relation was fairly known as the carrying capacity of *C. quinquefasciatus*, $K_c = K_{ss}$, we performed a restricted regression with a fixed intercept and observed the following relationship: *C. quinquefasciatus* = 320 - 0.39 * *D. magna* ($p < 0.001$). From this relationship, we estimated the population size of *D. magna* when *C. quinquefasciatus* was 0 and received $K_i = 831$ daphnids.

The competition coefficient $c_{ss,i}$, that describes the growth-limiting effects of *D. magna* on ss mosquitoes was inferred from the average population sizes of *D. magna*, $\widehat{K}_i$, and from *C. quinquefasciatus*, $\widehat{K}_c$, when both species developed together under non-toxic conditions. On average, the addition of $\widehat{K}_i = 430$ individuals of *D. magna* caused a reduction of $K_c - \widehat{K}_c = 133$ individuals of *C. quinquefasciatus*; therefore, $c_{ss,i}$ was calculated as $c_{ss,i} = \frac{K_c - \widehat{K}_c}{\widehat{N}_i} = 0.306$. Likewise, the competition coefficient for the effects of ss mosquitoes on *D. magna* was calculated as $c_{i,ss} = \frac{K_i - \widehat{K}_i}{\widehat{K}_c} = 2.118$.

Because $K_{ss} > K_i * c_{ss,i}$ and $K_i > K_{ss} * c_{i,ss}$, *C. quinquefasciatus* and *D. magna* were both stronger intraspecific than interspecific competitors; this leads to a stable coexistence of both species at population sizes of $\widehat{K}_c$ and $\widehat{K}_i$ as a result of partial niche separation [9, 10]. We inferred the degree of resource overlap between *D. magna* and ss mosquitoes under non-toxic conditions, $o_{ss,i}$, from $c_{i,ss}$ and $c_{ss,i}$: If the niches of both species fully overlap, the competition coefficients represent the ratio of their competitive strengths, $comp_{ss}$ and $comp_i$. *comp* corresponds to the area under the resource utilisation curve of a species (figure 5). Without niche separation the stronger competitor depletes the available resources to concentrations which do not support the survival of the weaker competitor [9, 11]; therefore$c_{ss,i} = \frac{1}{c_{i,ss}}$ and in equilibrium the weaker competitor becomes extinct. Niche separation reduces $c_{ss,i}$ and $c_{i,ss}$, depending on the degree of niche overlap $o_{ss,i}$: $c_{ss,i} = \frac{comp_i}{comp_{ss}} * o_{ss,i}$ and $c_{i,ss} = \frac{comp_{ss}}{comp_i} * o_{ss,i}$ with $o_{ss,i} < 1$. Setting $comp_{ss} = 1$ as a reference, the conversion leads to $o_{ss,i} = \sqrt{c_{ss,i} * c_{i,ss}} = 0.80$. With $o_{ss,i}$ known, we calculated $comp_i = \frac{c_{ss,i}}{o_{ss,i}} = 0.380$.

Because we hypothesised that the degree of niche overlap with *D. magna* differed for the different genotypes of *C. quinquefasciatus*, we estimated $o_{sr,i}$ and $o_{rr,i}$ in the same manner as described above for $comp_{rr}$ and $comp_{sr}$. $o_{rr,i}$ was considered to be the same under toxic and non-toxic conditions because rr mosquitoes were resistant and *D. magna* was not exposed to

the pesticide in our experiment. Therefore, we estimated $o_{ss,i}$ and $o_{sr,i}$ under toxic conditions as described above.

Model Sensitivity Analysis:

To study how the microevolutionary effects of predation or interspecific competition change with the model parameters, we ran the simulation with the parameter values estimated above and varied one or two of the values at the same time (figure 3). The effect size of predation or interspecific competition was expressed in % increase of the time required for the ss genotype to increase to > 99 % (under non-toxic conditions) or to decrease to < 1 % (under toxic conditions).

References

1. Berticat C, Boquien G, Raymond M, Chevillon C. 2002 Insecticide resistance genes induce a mating competition cost in *Culex pipiens* mosquitoes. *Genet Res* **79**, 41-47. (doi:10.1017/s001667230100547x)
2. Bourguet D, Pasteur N, Bisset J, Raymond M. 1996 Determination of ace.1 genotypes in single mosquitoes: toward an ecumenical biochemical test. *Pestic Biochem Physiol* **55**, 122-128. (doi:10.1006/pest.1996.0041)
3. OECD. 2004 OECD Guideline for the testing of chemicals, Section 2. In *Test No. 202: Daphnia sp acute immobilisation test*. Paris, FR, OECDpublishing. (doi:10.1787/20745761)
4. Foit K, Kaske O, Wahrendorf DS, Duquesne S, Liess M. 2012 Automated Nanocosm test system to assess the effects of stressors on two interacting populations. *Aquat Toxicol* **109**, 243-249. (doi:10.1016/j.aquatox.2011.09.013)
5. Foit K, Kaske O, Liess M. 2012 Competition increases toxicant sensitivity and delays the recovery of two interacting populations. *Aquat Toxicol* **106**, 25-31. (doi:10.1016/j.aquatox.2011.09.012)
6. Crawley MJ. 2007 *The R Book*. Chichester, UK, John Wiley & Sons Ltd.
7. Mulatti P, Ferguson HM, Bonfanti L, Montarsi F, Capelli G, Marangon S. 2014 Determinants of the population growth of the West Nile virus mosquito vector Culex pipiens in a repeatedly affected area in Italy. *Parasites & Vectors* **7**. (doi:10.1186/1756-3305-7-26)
8. Birch LC. 1948 The intrinsic rate of natural increae of an insect population. *J Anim Ecol* **17**, 15-26. (doi:10.2307/1605)
9. Begon M, Harper JL, Townsend CR. 2006 *Ecology: From Individuals to Ecosystems*, 4th edn, pp. 151-265. Oxford, UK, Blackwell Science Ltd.
10. Lotka AJ. 1932 The growth of mixed populations: two species competing for a common food supply. *Journal of the Washington Academy of Sciences* **22**, 461-469.
11. Tilman D. 1997 Mechanisms of plant competition. In *Plant Ecology* (ed. Crawley M.), 2nd edn, pp. 239-261. Oxford, UK, Blackwell Science Ltd.
