# Supplementary material for: Biotic interactions govern genetic adaptation to toxicants
Source: Proc Biol Sci. 2015 May 7;282(1806):20150071. doi: 10.1098/rspb.2015.0071 (PMC4426622; doi:10.1098/rspb.2015.0071)
Supplement: ESM Figures [file rspb20150071supp2.pdf]

Figure S1:

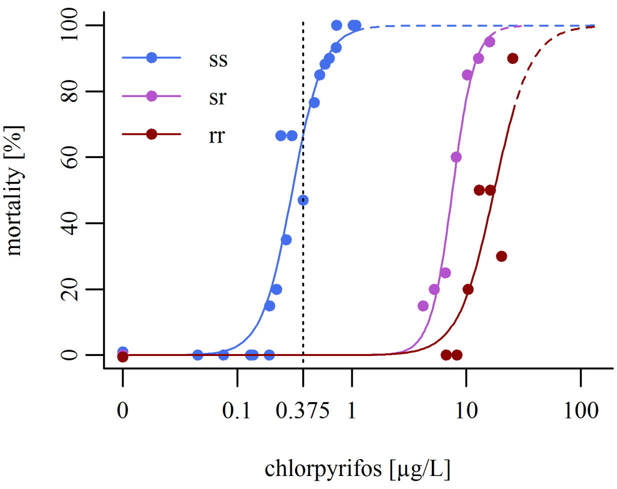

Figure S1. Contamination profiles of the organophosphorous pesticide chlorpyrifos for different *ace-1* genotypes in *Culex quinquefasciatus*. Groups of 5 first-instar larvae that were homozygous for the *ace-1*^{R} allele (rr), heterozygous (sr) or susceptible (ss) were contaminated in Elendt M4 medium without food. After 24 h, the larvae that did not move after disturbance with a blunt probe for five seconds were considered dead. Dashed lines represent extrapolated data. The dotted line indicates the concentration used for contamination in the main experiment.

Figure S2:

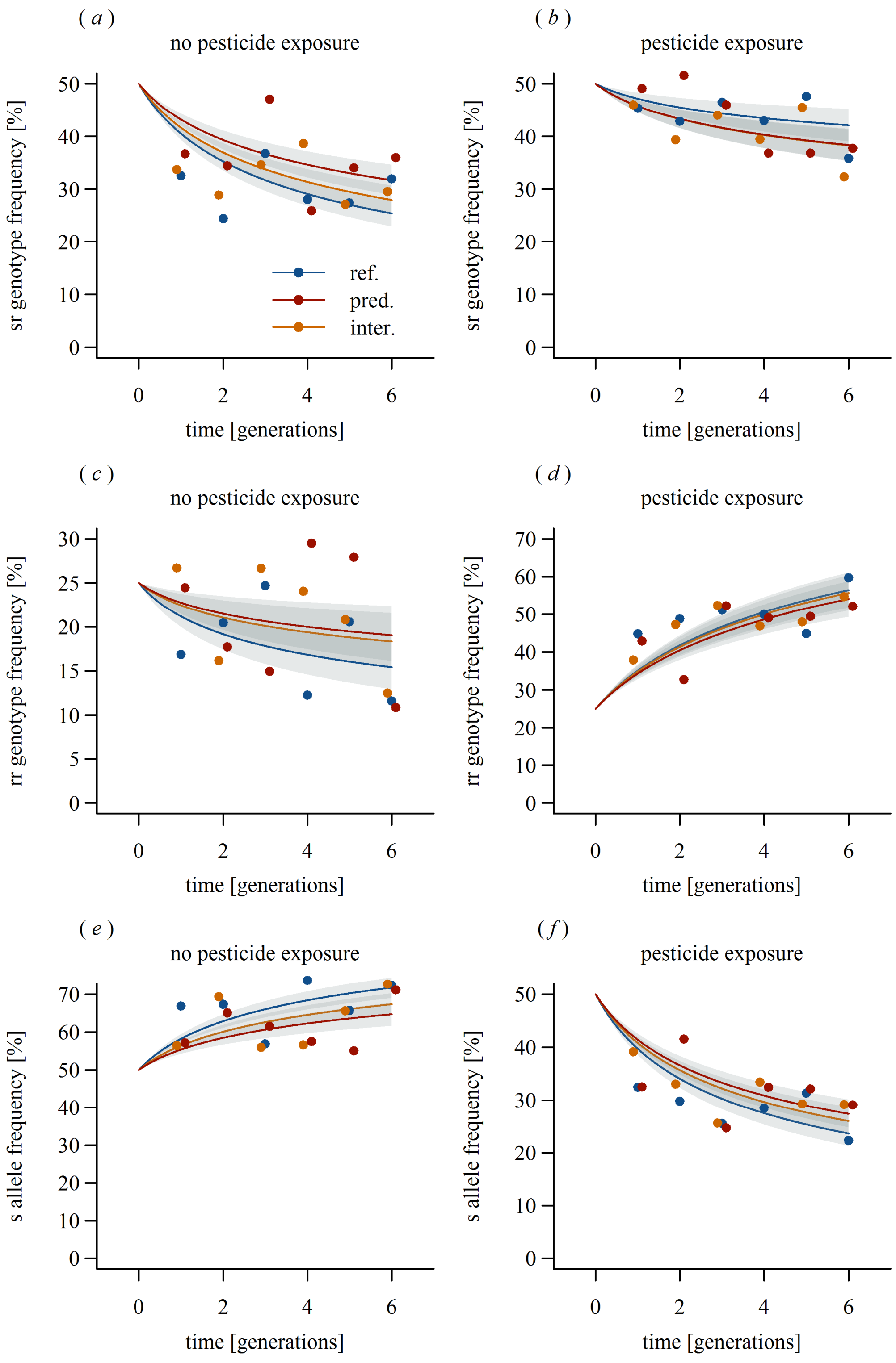

(a)
no pesticide exposure
sr genotype frequency [%]
ref.
pred.
inter.
time [generations]
(b)
pesticide exposure
sr genotype frequency [%]
time [generations]
(c)
no pesticide exposure
rr genotype frequency [%]
time [generations]
(d)
pesticide exposure
rr genotype frequency [%]
time [generations]
(e)
no pesticide exposure
s allele frequency [%]
time [generations]
(f)
pesticide exposure
s allele frequency [%]
time [generations]

Figure S2. (*a*) – (*b*) The effects of non-selective predation (pred.) and interspecific competition (inter.) on the heterozygous genotype frequency under toxic and non-toxic conditions compared to reference populations (ref.) without species interactions. (*c*) – (*d*) The effects on the resistant genotype frequency and (*e*) – (*f*) on the frequency of the susceptible allele. N = 4 for each group of populations. The points represent the mean values for each generation and were calculated using separate generalised linear models with a binomial error distribution for each generation. The lines represent the trend over all generations, which was analysed using a generalised linear mixed effects model with the log-transformed generations included as a covariate; the intercepts were fixed at the known initial genotype or allele frequency. The means ± SEM are reported.
