# Supplementary material for: Biotic interactions govern genetic adaptation to toxicants
Source: Proc Biol Sci. 2015 May 7;282(1806):20150071. doi: 10.1098/rspb.2015.0071 (PMC4426622; doi:10.1098/rspb.2015.0071)
Supplement: ESM Tables [file rspb20150071supp3.pdf]

Electronic Supporting Material:

Tables

Table S1. Analysis of deviance for the effects of species interactions and pesticide contamination on the larval density of *C. quinquefasciatus*. The generalised linear mixed effects model was fit by the maximum likelihood method (Laplace approximation) using a Gamma error distribution and an inverse link function. The Holm adjustment for multiple testing was applied to the contrasts of simple effects. The test cylinders were nested within the populations. The colons represent interactions. The asterisks indicate significant contrasts; *P<0.05, ***P<0.001.

**fixed effects (type II Wald χ^2 tests)**	**χ^2**	**d.f.**	**p**	
species interaction	22.39	2	< 0.001	***
contamination	5.07	1	0.02439	*
species interaction : contamination	34.09	2	< 0.001	***
non-toxic : ref. - pred.	1.2319	1	0.53408	
non-toxic : ref. - comp.	7.42	1	0.01933	*
toxic : ref. - pred.	0.06	1	0.811	
toxic : ref. - comp.	48.17	1	< 0.001	***
non-toxic - toxic : ref. - pred.	30.953	1	< 0.001	***
**random effects**	**variance**	**std.dev.**		
intercept | population	< 0.01	< 0.01		
intercept | test cylinder : population	< 0.01	< 0.01		
residual	0.51	0.71		

deviance = 2461.6; AIC = 2479.6; BIC = 2512.6; log-likelihood. = -1230.8; residual d.f. = 279
observations = 288; groups = 48 (test cylinder : population), 24 (population)

Table S2. Analysis of deviance for the effects of pesticide contamination and species interactions on the change of the homozygous susceptible genotype frequency over time. Time was measured in generation times and log-transformed to improve the model fit. The initial genotype frequency was known and fixed at 25 %; therefore the main effects were excluded. The generalised linear mixed effects model was fit by the maximum likelihood method (Laplace approximation), using a binomial error distribution and a logit link function. The Holm adjustment for multiple testing was applied to the contrasts of simple effects. The colons represent interactions. The asterisks indicate significant contrasts; *P<0.05, **P<0.01, ***P<0.001.

**fixed effects (type II Wald χ^2 tests)**	**χ^2**	**d.f.**	**p**	
log(generation + 1) : contamination	493.52	2	< 0.001	***
log(generation + 1) : species interaction	0.23	2	0.891	
log(generation + 1) : contamination : species inter.	16.51	2	< 0.001	***
log(generation + 1) : non-toxic : ref. - pred.	5.29	1	0.043	*
log(generation + 1) : non-toxic : ref. - comp.	1.83	1	0.176	
log(generation + 1) : toxic : ref. - pred.	10.27	1	0.005	**
log(generation + 1) : toxic : ref. - comp.	7.51	1	0.018	*
**random effects**	**variance**	**std.dev.**		
log(generation + 1) | population	0.012	0.108		

deviance = 861.1; AIC = 875.1; BIC = 895.9; log-likelihood. = -430.5; residual d.f. = 137; observations = 144; groups = 24 (population)

Table S3. Analysis of deviance for the effects of sex, genotype, species interactions, pesticide contamination and time of the experiment (expressed in generations) on the wing length of adult *C. quinquefasciatus*. Wing length was measured from the first, second and sixth generations, and the generations were log-transformed to improve the model fit. The linear mixed effects model was fit by the restricted maximum likelihood method. The Holm adjustment for multiple testing was applied to the contrasts of simple effects. The colons represent interactions. The asterisks indicate significant contrasts; . < 0.1; *P<0.05, **P<0.01, ***P<0.001.

fixed effects (type II Wald χ^2 tests)	χ^2	d.f.	p	
sex	2277.42	1	< 0.001	***
genotype	3.32	2	0.190	
species interaction	13.72	2	0.001	**
contamination	3.40	1	0.065	.
log(generation + 1)	19.11	1	< 0.001	***
sex : species interaction	8.30	2	0.016	*
sex : contamination	0.01	1	0.929	
species interaction : contamination	1.58	2	0.453	
genotype : contamination	5.30	2	0.071	.
log(generation + 1) : genotype	2.66	2	0.265	
log(generation + 1) : contamination	0.13	1	0.717	
log(generation + 1) : species int.	6.28	2	0.043	*
log(generation + 1) : ref.	18.34	1	< 0.001	***
log(generation + 1) : pred.	15.67	1	< 0.001	***
log(generation + 1) : comp.	1.23	1	0.268	
sex : species interaction : contamination	9.01	2	0.011	*
log(generation + 1) : genotype : contamination	20.26	2	< 0.001	***
log(generation + 1) : ss - sr : non-toxic - toxic	5.06	1	0.024	*
log(generation + 1) : ss - rr : non-toxic - toxic	19.62	1	< 0.001	***
log(generation + 1) : sr - rr : non-toxic - toxic	8.36	1	0.008	**
**random effects**	**variance**	**std.dev.**		
intercept | population	12418.00	111.44		
log(generation + 1) | population	4767.00	69.04		
residual	56928.00	238.60		
REML criterion at convergence = 41781.11; observations = 3039; groups = 24 (population)				

Table S4. Life table for the calculation of the intrinsic growth rate of *Daphnia magna*. The survival and fecundity after 21 days were estimated from observations during the experiment and the colony strains. x = age of a cohort (in weeks and in generation times of *C. quinquefasciatus* = 4 weeks); l_x = probability of survival to x; m_x = female offspring per female produced in x.

x [days]	x [generations]	l_x	m_x	l_x m_x
7	0.25	1.000	12.120	12.120
14	0.50	0.890	5.520	4.913
21	0.75	0.821	2.000	1.642

Table S5. Estimated model parameters and start values for the simulation of the experimental conditions. See text for details. * Competitive strength *comp* and the degree of resource overlap, *o*, between the interspecific competitors were not directly included in the model but were used to calculate the competition coefficients *c*. ** The dominance of the fitness costs for a given parameter was not directly included in the model but was used to calculate *r*, *K*, and *c* for the sr genotype.

parameter	non-toxic conditions	toxic conditions
r_{ss}	2.00	1.92
r_{sr}	1.92	1.92
r_{rr}	1.92	1.92
r_i	10.08	10.08
K_{ss}	320.00	218.00
K_{sr}	272.00	272.00
K_{rr}	272.00	272.00
K_i	831.00	831.00
$c_{ss,sr}$	0.99	1.14
$c_{ss,rr}$	0.99	1.14
$c_{ss,i}$	0.30	0.28
$c_{sr,ss}$	1.01	0.88
$c_{sr,rr}$	1.00	1.00
$c_{sr,i}$	0.29	0.29
$c_{rr,ss}$	1.01	0.88
$c_{rr,sr}$	1.00	1.00
$c_{rr,i}$	0.29	0.29
$c_{i,ss}$	2.11	1.48
$c_{i,sr}$	1.98	2.00
$c_{i,rr}$	1.98	2.00
a_{ss}	0.67	0.67
a_{sr}	0.67	0.67
a_{rr}	0.67	0.67
a_i	0.00	0.00
f_{ss}	0.00	0.00
f_{sr}	0.00	0.00
f_{rr}	0.00	0.00

q	0.00	0.00
$comp_{ss}$ *	1.00	0.87
$comp_{sr}$ *	0.99	0.99
$comp_{rr}$ *	0.99	0.99
$comp_{i}$ *	0.38	0.38
$o_{ss,i}$ *	0.80	0.65
$o_{sr,i}$ *	0.76	0.77
$o_{rr,i}$ *	0.76	0.77
dominance.r **	1.00	0.00
dominance.K **	1.00	0.00
dominance.o **	1.00	0.00
dominance.comp **	1.00	0.00
$N.start_{ss}$	50.00	50.00
$N.start_{sr}$	100.00	100.00
$N.start_{rr}$	50.00	50.00
$N.start_{p}$	1.00	1.00
$N.start_{i}$	200.00	200.00
